# Tuberculosis treatment and cytotoxic immune signatures: a longitudinal study

**DOI:** 10.1016/j.jctube.2026.100626

**Published:** 2026-06-23

**Authors:** Catherine Sulca, Alessia Piamonte, María A. Quispe-Ricalde, Sandra Delgado, Sandra Palma Albornoz, Iskra Tuero

**Affiliations:** a Facultad de Medicina Humana, Universidad de San Martin de Porres, Lima, Peru; b Laboratorios de Investigación y Desarrollo, Facultad de Ciencias e Ingeniería, Universidad Peruana Cayetano Heredia. Lima, Peru; c Departamento de Biología, Universidad Nacional San Antonio Abad del Cusco, Cusco, Peru; d Hospital Central de la Fuerza Aérea del Perú, Lima, Peru.

**Keywords:** Tuberculosis, Mycobacterium, Ag85A, Cytotoxicity, Antibodies

## Abstract

**Background:**

Tuberculosis (TB) remains a leading cause of global mortality. Current tools for monitoring therapeutic efficacy are suboptimal, creating an urgent need for novel strategies. While emerging evidence suggests that immune biomarkers could fill this gap, our understanding of antibody dynamics during TB treatment remains limited. Beyond neutralization, antibodies play a critical role in the immune response by modulating inflammation and mediating the clearance of infected cells.

**Methods:**

In a longitudinal cohort of active TB patients, we analyzed Ag85A-specific antibody-dependent cellular cytotoxicity (ADCC) and perforin-expressing NK cells via flow cytometry. Ag85A-specific IgG subclasses were measured by ELISA to correlate humoral architecture with cytotoxic potency.

**Results:**

The analysis revealed a significant increase in Ag85A-specific antibodies capable of mediating cytotoxic activity, which was associated with elevated levels of NK cells producing perforin upon treatment completion. Furthermore, ADCC activity correlates with *Mtb*-specific IgG subclasses and treatment time duration. Notably, a longitudinal decline in Ag85A-specific IgG3 levels emerged as a distinct signature of bacterial clearance and clinical resolution, suggesting a transition from acute inflammatory responses toward a resolved immune profile characterized by sustained cytotoxic potential.

**Conclusions:**

Clinical resolution of TB is characterized by the potentiation of the ADCC-NK cell axis. The reduction in IgG3 levels reflects a shifting inflammatory milieu, while enhanced cellular cytotoxicity serves as a primary immunological hallmark and a potential biomarker for treatment monitoring.

## Highlights

• TB cured patients show enhanced Ag85A-specific ADCC activity.
• Increased perforin-producing NK cells correlate with ADCC.
• Ag85A-specific IgG3 levels decrease significantly upon bacterial clearance

## 1 Background

The Bacille Calmette-Guérin (BCG) vaccine shows limited efficacy against adult pulmonary tuberculosis (TB), highlighting a critical gap in our understanding of protective immunity. This lack in knowledge directly translates into a global clinical crisis. Despite standardized treatments, active TB (ATB) remains a public health problem with over 10 million new cases annually [1], [2]. The current monitoring tools for treatment follow-up have low sensitivity and specificity. In recent years, host immune biomarkers are increasingly recognized as vital tools for monitoring TB treatment efficacy, predicting clinical outcomes, and identifying risks of relapse or adverse effects [3].

The immunological paradigm in TB has long been governed by the T cell-mediated Th1 response and IFN-γ production, viewed as the canonical axis for macrophage activation and intracellular bacterial control [4]. However, vaccines designed to induce a strong Th1 response have failed to elicit effective protection. Emerging evidences now highlights a more complex landscape where B cells, natural killers (NK) cells, and specific antibody signatures act as pivotal orchestrators of the host defense [5], [6].

While historically overlooked, humoral immunity is now recognized as a critical component in the defense against intracellular pathogens. Evidence from malaria [7], SARS-CoV-2 [8], influenza [9], Ebola [10]**,** and HIV [11] has established that IgG antibodies and their subclasses exert significant control over pathogen dynamics during infection. In TB, *Mtb*-specific antibodies bridge adaptive and innate immunity via their Fc regions engaging cell Fc receptors (FcR) to trigger potent antimicrobial mechanisms. Theses pathways including antibody-mediated cellular cytotoxicity (ADCC), antibody-dependent cellular phagocytosis (ADCP), or complement-dependent cytotoxicity (CDC), could eliminate *Mtb-*infected cells [12], [13], [14]. NK cells drive ADCC via FcγRIIIa (CD16) signaling [15]. Interestingly, ATB is characterized by impaired *Mtb*-specific ADCC and NK cell activation relative to latent TB [12]. TB treatment further modulates this axis, triggering phenotypic shifts in, NK cells degranulation and IFN-γ production [16], alongside transient decreases in *Mtb*-specific IgG4 and altered glycosylation [17] suggesting that the humoral- cellular axis is highly dynamic and sensitive to bacterial load.

In this longitudinally study, we elucidated the dynamic of Ag85A-specific antibodies ADCC and NK cells effector function throughout TB treatment. By integrating *Mtb*-specific IgG subclass profiling with ADCC assays and perforin producing NK cell quantification, we decoded the synergistic interplay between humoral and cellular effectors. Our multidimensional analysis identifies distinct immune signatures that may serve as a foundation for robust biomarkers to longitudinally monitor therapeutic efficacy in tuberculosis.

## 2 Material and methods

### 2.1 Study participants

We conducted a longitudinal cohort study of 20 patients (18–65 years) with ATB, diagnosed via clinical evaluation, imaging and microbiology (sputum smear test (SST), culture (SCT) or GeneXpert). Exclusion criteria included previous anti-TB treatment, confirmed immune deficiency disease, HIV, comorbidities, pregnancy, extrapulmonary, latent, or resistant TB. All participants provided written informed consent prior to enrollment.

### 2.2 Samples

Whole blood was collected via venipuncture (K2-EDTA) at baseline (T0- pre-treatment), two months (T2), and upon treatment completion (T6; six-months).

The plasma obtained was stored at −20 °C and heat-inactivated prior to use in ADCC and ELISA assays.

### 2.3 Antigens and cell line

Recombinant Ag85A from *Mtb* (Cat # NR-49427), Spike Glycoprotein Receptor Binding Domain (RBD) from SARS-CoV2 (Cat# NR-53800), and CEM-NKR cell line were obtained from Biodefense and Emerging Infections Research Resources Repository (BEI Resources, NIAID, NIH).

### 2.4 Mtb-specific-antibody measurements

Ag85A-specific IgG subclasses were measured by ELISA [18]. Maxisorp plates were coated with Ag85A (1 μg/mL) or of Spike Glycoprotein RBD (0.5 μg/mL) overnight at 4 °C. After blocking with 1% BSA, samples (diluted 1:5 for IgG1- IgG3, and 1:20 for IgG4) were incubated for 1.5 h. Detection utilized HRP-conjugated anti-human IgG1, IgG2 and IgG4 (1:500) or biotinylated anti-human IgG3 (1:1500) followed by streptavidin-HRP (dilution 1:1500). Reactions were developed with TMBs, stopped with 2 M H_2_SO_4_ and read at 450 nm. Optical density (O.D.) values were background-corrected by subtracting negative control wells. Spike Glycoprotein Receptor Binding Domain (RBD) was used as heterologous antigen control to assess antibody specificity and cross-reactivity.

ADCC was quantified as previously described [19]. CEM-NKR target cells were dual-stained with PKH26 and CSFE, pulsed with recombinant Ag85A and opsonized with patient plasma (1:40) for 30 min at 37 °C. Healthy donor PBMC (effectors) were added in an effector-to-target (E:T) ratio of 50:1. After 4 h of co-culture, ADCC was assessed by quantifying the loss of CFSE in 2000 PKH^+^ target events using a FACS Canto II (BD Biosciences). Cytolysis was quantified as the PKH-26^+^CFSE^−^ population using FlowJoV10.9 (TreeStar Inc). Data were normalized to BSA-CEM-NKR-coated negative controls and expressed as fold-increase in ADCC activity. To minimize inter-assay variability, matched longitudinal samples (T0, T2, T6) were processed in a single batch and assayed in duplicate for all assays.

### 2.5 Natural killer cells analysis

In 200 μL whole blood, NK cells were phenotyped via surface-staining with a combination of antibodies: anti-human CD3- Pacblue (UCHT1), CD8-FITC (STK1) and CD56-BV510 (HCD56) [20]. After erythrocytes lysis and washing, cells were fixed and permeabilized using a Perm/fix solution (BD Biosciences) for intracellular staining with Perforin-PE (dG9) in 2% FBS. At least 500,000 events were acquired on a FACS CANTO II flow cytometer and analyzed using FlowJo V10.9. NK cells were defined as CD3^−^CD8^−^CD56^+^ (to exclude NKT cells) and gating thresholds were established using Fluorescence Minus One (FMO) control.

### 2.6 Statistical analysis

Statistical analyses were performed in GraphPad Prism (v.10.4.1), RStudio (v.2025.09) and MATLAB (v.2024b). Given the non-Gaussian distribution and repeated measures design, longitudinal trajectories (Baseline/T0, T2, and T6) were analyzed via Friedman test with Nemenyi post hoc correction for multiple comparisons. Given the non-parametric distribution of the dataset, statistical significance was assessed using the two-tailed Mann-Whitney U test to evaluate potential sex-specific differences in TB-specific subclass levels, NK cell frequencies, and cytotoxic activity. Bivariate associations were determined using Spearman's rank correlation coefficient (ρ). Multivariate structure was explored through Principal Component Analysis (PCA), while chord diagrams (R package circlize) visualized correlations networks, with chords widths scaled to Spearman coefficients.

## 3 Results

### 3.1 Study populations and clinical follow-up

We longitudinally evaluated 20 patients with active pulmonary TB over six months. Clinical and microbiological assessments, including smear sputum test (SST) for bacterial clearance (Table 1), were performed across treatment.

**Table 1:** Cohort of TB patients (*n* = 20).

**Demographic**			
**Age, y**	34.1 ± 15.8^§^		
**Sex:**			
Male	16 (80%)		
Female	4 (20%)		
**Laboratory test**			
**SST**	**T0**	**T2**	**T6**
*Negative*	2 (10.0%)*	18 (90%)	20 (100%^)¥^
*(+)*	12 (60.0%)	1 (5%)	0 (0%)
*(++)*	2 (10.0)	1 (5%)	0 (0%)
*(+++)*	4 (20.0%)	0 (0%)	0 (0%)
**SCT**	**T0**	**T2**	**T6**
Positive	11	–	–
Negative	5	–	6
Not available	4	20	14^¥^
**GeneXpert MTB/RIF:**			
Susceptible	15 (75%)		
Resistance	–		
Not determined	5 (25%)		

T0: baseline/before initiating treatment; T2: after 2 months of treatment; T6: 6 months completed treatment. SST = smear sputum test. *Clinical diagnosis. ^§^mean ± SD. GeneXpert MTB/RIF results (T0) are not complete for all patients because not performed or not enough sample and the status of these patients was based on SST or SCT results. ^¥^The cured status was established by pulmonologists using clinical and microbiological assessments.

At baseline, 90% (18/20) of patients were smear-positive, with 60% (12/20) exhibiting <2+, 30% (6/20) had ≥2+. Two patients (2/20, 10%) presented with negative SST results (but with clinical diagnosis). By T2, 10% (2/20) of patients remained ≤2+. By the end of treatment (T6), 100% (20/20) of patients showed negative SST results, with successful treatment completion, and clinical cure.

### 3.2 Enhanced Ag85A-specific ADCC activity in cured TB patients

Fc-mediated antibody effector functions, including cytotoxicity, phagocytosis, and others are pivotal in the immune response against *M. tuberculosis* [6], [12], [17]. We longitudinally characterized Ag85A-specific antibody functional shifts during TB treatment utilizing flow cytometry (Supplemental file 1) to assess ADCC activity in plasma samples.

**Fig. 1:**
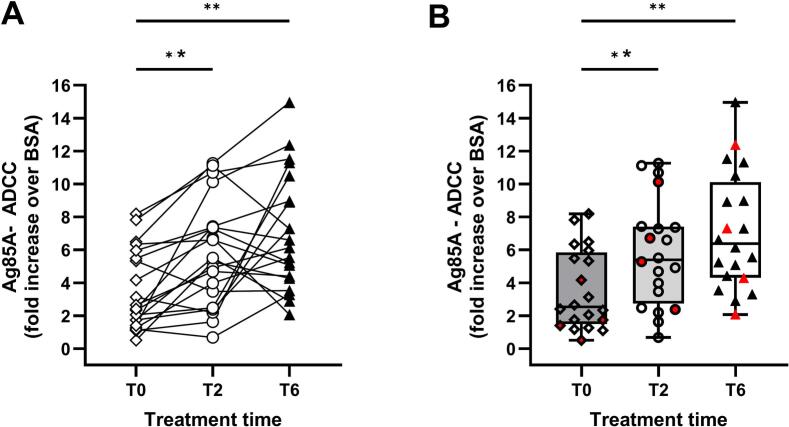
**Ag85A-specific ADCC evolution during anti-TB treatment**. Plasma samples were collected from active TB patients at baseline (T0), T2 (two months), and T6 (six months). **(A)** Longitudinal trajectories of Ag85A-specific ADCC scores. **(B**) Box plots (median and range) for each time point. Data for female participants highlighted in red color. Statistical analysis was performed using the non-parametric Friedman test, followed by post hoc Nemenyi testing. **p < 0.01 indicate significant differences between time points.

Ag85A specific-ADCC activity increased significantly following therapy initiation (Figs. 1A-B). Elevated levels were observed after 2 months (T2) in 85% of patients (17/20, *p* = 0.0072) and remained sustained through treatment completion (T6) in 75% (15/20, *p* = 0.0075) relative to baseline (T0). These data link the clinical recovery to a humoral shift toward potent cytotoxic effector functions. To evaluate whether patient demographics confounded the observed immune kinetics, we stratified our cohort by sex. As illustrated in Supplemental File 2A, a comparative analysis revealed no statistically significant differences between male and female patients regarding cytotoxic activity.

### 3.3 Ag85A-specific IgG3 levels decrease during anti- TB treatment

**Fig. 2:**
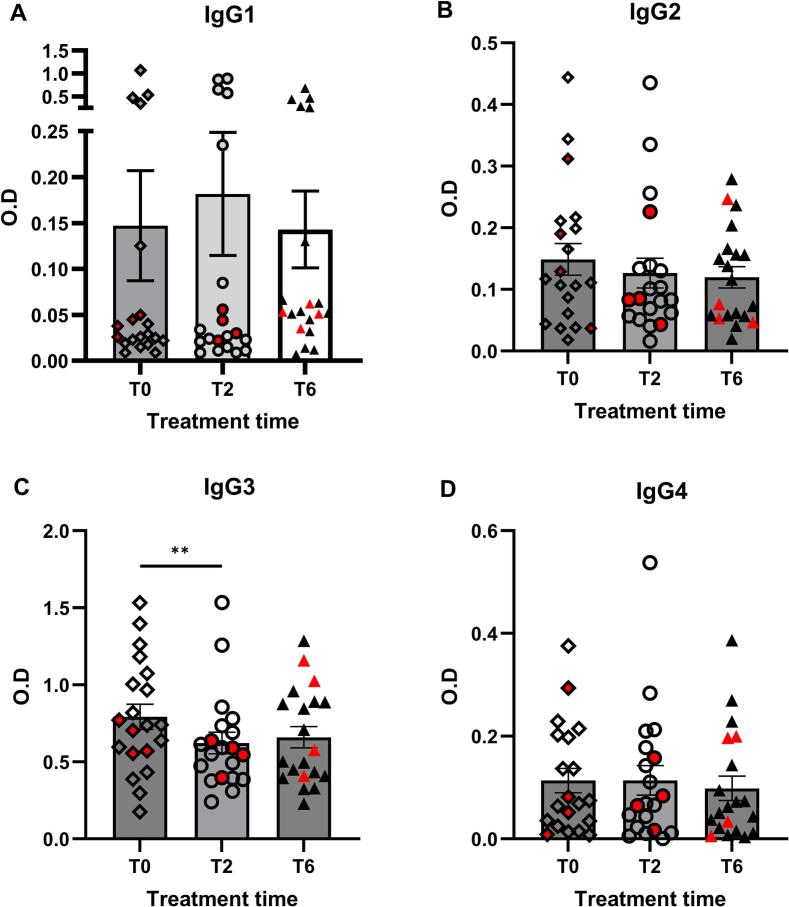
**Longitudinal dynamic of Ag85A-specific IgG subclasses across TB treatment**. Plasma levels of Ag85A-specific IgG1 **(A)**, IgG2 **(B)**, IgG3 **(C)**, and IgG4 **(D)** were measured at baseline (T0), T2 and T6. The y-axis represents the optical density (O·D) values. Results are expressed as mean ± SEM. Data for female participants highlighted in red color. Statistical significance was determined using the non-parametric Friedman test and the post hoc statistical Nemenyi test, **p < 0.01 indicates significant differences between treatment times.

The enhanced ADCC prompted longitudinal profiling of Ag85A-specific IgG subclasses to identify correlates of clinical resolution (Fig. 2).

While IgG1, IgG2, and IgG4 levels remained stable throughout therapy (Figs. 2A, B, D), IgG3 significantly declined by T2 in 80% of patients (16/20, *p* = 0.0075) compared to baseline (Fig. 2C).

To confirm antigen specificity, recombinant Spike Glycoprotein Receptor Binding Domain (RBD) was included as a heterologous control (Supplemental file 2). Unlike the stable Ag85A profiles, Spike-RBD-specific IgG4 decreases (*p* < 0.05) over six months, underscoring the divergent kinetic of *Mtb*-specific versus unrelated humoral antigen responses during TB recovery. No statistically significant differences were observed between male and female patients regarding levels of Ag85A and Spike-RBD specific IgG subclasses (Supplemental File 2B-I).

### 3.4 Immune correlates of Ag85A-specific humoral responses

**Fig. 3:**
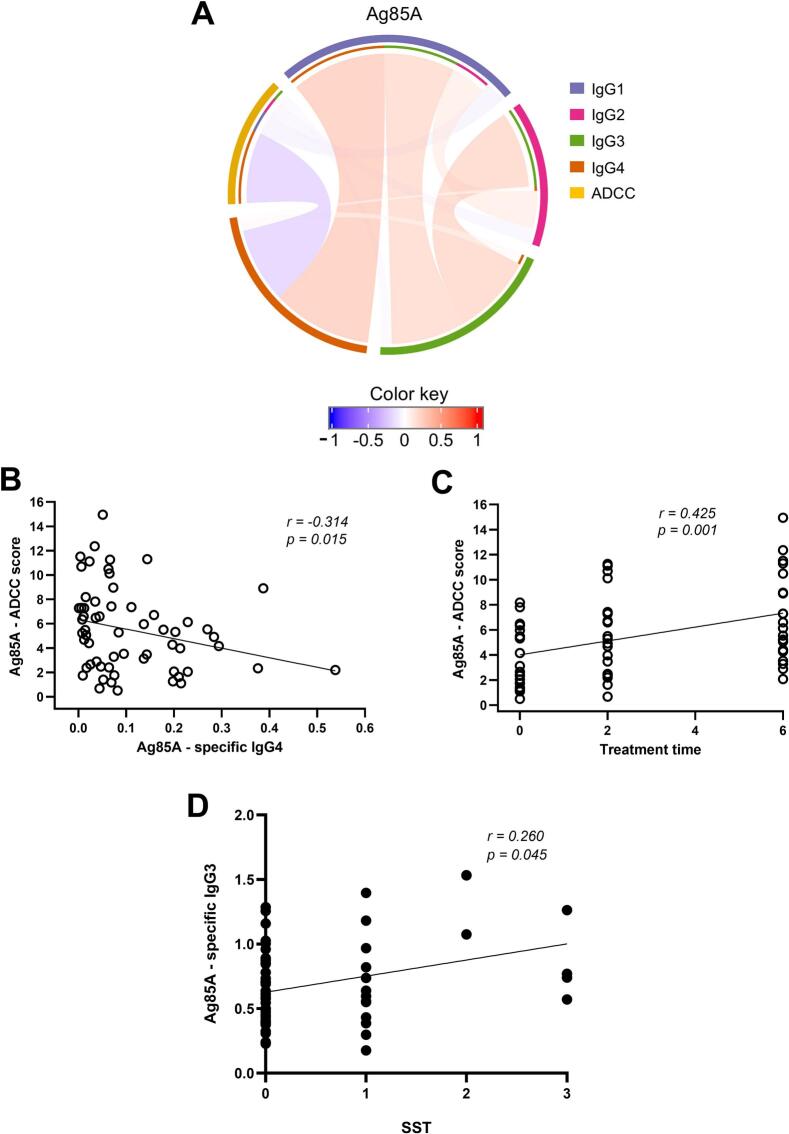
**Relationships among *Mtb* antigen-specific antibody subclasses and antibody-dependent cellular cytotoxicity (ADCC).** Chord diagrams illustrate the relationship between Ag85A-specific antibody features. Networks were constructed using Spearman correlation analyses to capture both the strength and direction of associations among immune parameters. The width and connectivity of arcs represent the magnitude and direction of significant associations. The colour scale reflects the Spearman correlation rho (r), ranging from strongly negative (purple, r ≈ −1) to strongly positive (red, r ≈ +1). Chord diagrams among *Mtb*-specific IgG1–IgG4 levels and ADCC activity **(A)**, individual correlation plots of ADCC score and Ag85A-specific IgG4 (**B**), and treatment time **(C)** and Ag85A-specific IgG3 and SST **(D)** are shown. In **(D)**, the SST number 0 = negative SST; 1 = 1 +; 2 = 2+; 3 = 3+. Only significant correlations are shown (*p* < 0.05). (For interpretation of the references to colour in this figure legend, the reader is referred to the web version of this article.)

Since IgG1 and IgG3 primarily drive antibody effector functions [6], [21], we correlated Ag-85-specific ADCC activity with IgG subclass, and treatment duration, as well as Ag85A-specific subclasses and SST to elucidate the relationship between enhanced cytotoxicity and the evolving humoral profile (Fig.3).

A chord diagram mapped the relationship between *Mtb*-specific subclasses, and ADCC activity (Fig. 3A). IgG1 positively correlated with both IgG3 (*r* = 0.316, *p* = 0.0140) and IgG4 (*r* = 0.418, *p* = 0.0010), but lacked direct association with ADCC activity (Fig. 3A), whereas, IgG4 modestly correlated negatively with ADCC (*r* = −0.31, *p* = 0.0150) (Fig. 3A and B).

TB infection and anti-TB treatment drives humoral remodeling [6], [12], [13], [14], [17]. We found that treatment duration modestly correlated positively with Ag85A-specific ADCC (*r* = 0.425, p = 0.001; Fig. 3C), marking a shift toward cytotoxic effector functions. Notably, IgG3 level modestly positively correlated with bacterial burden (SST) (*r* = 0.260, *p* = 0.045; Fig. 3D), reinforcing the potential of IgG3 as a surrogate for *Mtb* persistence.

We next evaluated age as a potential driver of immunological heterogeneity within our cohort. Association analyses confirmed that the longitudinal trajectories of the monitored immune parameters were entirely unaltered by patient age (Supplemental Table 1), further validating the robustness of the identified signature against baseline demographic variance.

### 3.5 NK cells producing perforin are associated with cytotoxic activities mediated by antibodies during TB treatment

NK cells exert potent antimicrobial activity via ADCC and the targeted release of cytolytic machinery, including perforin and granzymes [15].

**Fig. 4:**
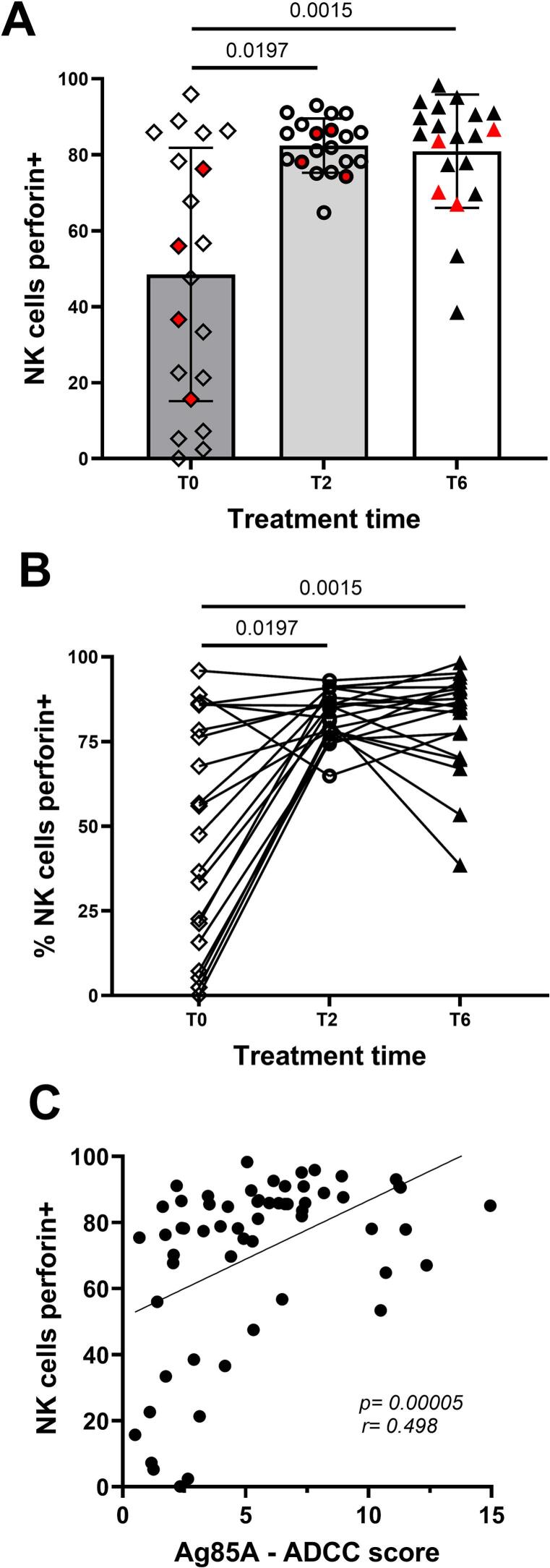
**Dynamic of NK cells producing perforin throughout anti-TB treatment.** Frequency of perforin^+^ NK cells at baseline, T2 and T6 **(A).** Longitudinal trajectories of individual patient data. Box plots representing median and range across the three treatment intervals **(B)**. Data are expressed as mean +/− SEM. Statistical significance between time points was assessed via Friedman test with Nemenyi post-hoc analysis. **(C)** Scatter plot between perforin-producing NK cells (CD3^−^CD8^−^CD56^+^Perforin^+^) and Ag85A-specific ADCC scores across all timepoints. Data for female participants highlighted in red color. Statistical association was determined using Spearman's rank correlation; linear regression trend (solid line). Spearman's rho (r) and two-tailed *p*-values are indicated within the plot.

To determine the cellular drivers of therapy-induced ADCC, we longitudinally profiled the CD3^−^CD8^−^CD56^+^ NK cell compartment and its cytolytic effector potential by quantifying perforin expression by flow cytometry (Supplemental file 4 and Fig.4).

TB therapy induced a progressive expansion of the perforin-producing NK cell (Fig. 4A-B), increasing in 80% of patients by T2 (16/20, *p* = 0.0197) and reached 90% by T6 (18/20, *p* = 0.0015). This progressive increase in perforin-producing NK cells suggest that clinical recovery facilitates the innate cytotoxic effectors.

No statistically significant differences between male and female patients regarding levels of NK cell frequencies (Supplemental file 2J).

To bridge cellular capacity and humoral function, we correlated NK cell activation with ADCC. As expected, a positive correlation (*r* = 0.498, *p* = 0.00005) was observed between perforin-producing NK cell frequency and cytotoxicity score (Fig. 4C), indicating that treatment restores the NK cell cytolytic machinery that directly drives the enhanced ADCC necessary for potentially controlling *Mtb* during clinical resolution.

### 3.6 Differential immune profile across TB treatment

**Fig. 5:**
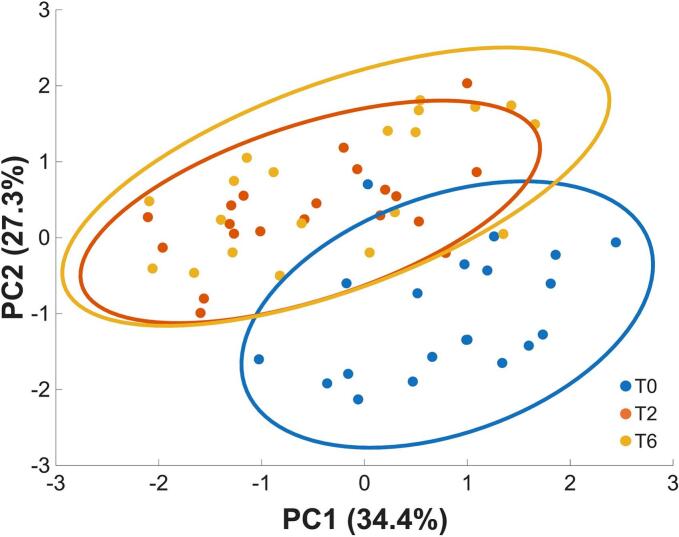
**Multivariate integration of Ag85A-specific immune signature and clinical parameters.** PCA score plot demonstrating the longitudinal immunological trajectory of TB patients (*n* = 20) at baseline (T0, blue circle), T2 (orange circle), and T6 (yellow circle). PC1 and PC2 axes denote explained variance, with ellipses representing 95% confidence interval for each group. (For interpretation of the references to colour in this figure legend, the reader is referred to the web version of this article.)

Principal component analysis (Fig. 5) integrated Ag85A-specific humoral responses, ADCC, and bacterial load, capturing the evolving immunological landscape. The analysis revealed a distinct transition from baseline (T0) toward stabilized clustering at T2 and T6 ((PC1: 34.4%, PC2: 27.3%), signaling a convergence of the immune profile during recovery.

## 4 Discussion

In TB treatment, limited information is available regarding the contribution of antibodies to controlling *Mtb* infection. Therefore, we characterized the longitudinal evolution of *Mtb*-specific antibody reveling that clinical cure is associated with a profound functional shift in the humoral response.

The Ag85 complex (A, B, and C) is a critical 30–32 kDa virulence factor essential for cell wall synthesis. Ag85A, a dominant immunogen highly expressed during active replication, remains a primary vaccine candidate [22], [23], [24]. However, its early-phase dominance highlights the need for antibody-based strategies to account for dynamic antigenic shifts across the *Mtb* infection cycle [25]. Our data demonstrated that TB resolution is accompanied by an enhanced capacity of Ag85A-specific antibodies to mediate ADCC. This response was associated to a significant increase in perforin-producing NK cells at the end of treatment. This suggests a potential functional restoration of the innate immune compartment, consistent with reports indicating that active TB induce NK cells exhaustion with reduced frequency, lower IFN-γ expression, and impaired degranulation [16], [26].

The enhanced ability of antibodies to mediate high ADCC in cured patients may be related to qualitative shift in antibody properties such as glycosylation patterns [6], [12], [27], isotype distribution [28], and B cell phenotype [29] driven by reduction in antigen load during *Mtb* clearance [25]. Interestingly, some patients maintained high perforin-producing NK cells levels from baseline, highlighting the impact of individual genetic backgrounds, or underlying inflammatory states on immune recovery [15], [16], [30] that should be considered for future analysis.

Among the IgG subclasses, IgG1 and IgG3 subclasses are the most efficient mediators of ADCC due to their high affinity for Fc receptors (FcγR) [21]. In our study a significant reduction in Ag85A-specific IgG3 levels was observed two months after starting treatment (T2). Given that IgG3 has a short-life and its expression is highly dependent on continuous antigenic stimulation [21]. Sustained IgG3 production may reflect persistent circulating antigen during therapy [31]. In the same line, high Ag85A-specific IgG3 levels were associated with the presence of bacilli in sputum, supporting the notion that short-lived cytophilic antibodies may serve as markers of active infection [32], [33]. A dual role for IgG3 as both a biomarker and a functional mediator of immune defense has been proposed, based on the association between elevated *Mtb*-specific IgG3 titers and protection against recurrent infection [34].

Notably, while Ag85-specific IgG3 levels decreased, at 2 months of treatment, Ag85A-specific ADCC remained elevated through treatment. This apparent discrepancy may be explained by Fc- glycosylation remodeling due changes in the inflammatory status [32], [35]. Glycosylation, a post-translational modification, can profoundly affect antibody structure, stability, and effector functions. Grace et al. 2021 described a distinct Fc-glycosylation profile in treated TB patients, characterized by reduced inflammatory Fc glycans and lower PPD-specific IgG4 titers. These findings suggest that antibody glycosylation and subclasses distribution may serve as a potential indicator of treatment success [17]. In line with these findings, our data indicate that Ag85A-specific IgG3 may undergo glycosylation remodeling, thereby modulating FcR affinity and dictating ADCC functionality [36]. Additionally, the maintenance of Ag85A-specific IgG1 levels across TB treatment likely provides a stable baseline for recruiting immune cells, compensating for the reduction in IgG3. Importantly, the observed decline in *Mtb*-specific IgG3 reflect a pathogen-specific clearance, rather than a systemic artifact from COVID-19 vaccinations. Although vaccination timelines were unrecorded, longitudinal anti-Spike RBD IgG trajectories remained flat across all timepoints for each participant. Because acute immunization characteristically triggers sharp, transient antibody spikes followed by a contraction phase [37], this lack of intra-individual fluctuation confirms the absence of recent immunizing events during the study window, validating the use of anti-Spike antibodies as an internal control. The striking kinetic divergence between pathogen-specific antibodies underscores how antigen persistence and pathogen architecture distinctly shape the humoral landscape. While the elevated anti-*Mtb* IgG3 pool is maintained by short-lived, active peripheral plasmablasts [38] fueled by persistent inflammation [[32], [33], [34], [35]], the Spike-RBD IgG3 pool represents a homeostatic memory baseline sustained by antigen-independent, bone-marrow-resident long-lived plasma cells [39]. Also, SARS-CoV2 RBD-specific responses are dominated by IgG1 and IgG3 in acute infection, but IgG1 predominates in most cohorts [40]. Upon antibiotic-mediated *Mtb* clearance, the loss of inflammation halts short-lived plasmablast activity [38], causing a rapid contraction of *Mtb*-specific due to its short half-life. Conversely, the viral-specific antibody reservoir remains unaffected by anti-TB therapy, validating its utility as a stable baseline control.

In contrast to the protective role of IgG1 and IgG3, IgG2 and IgG4 exhibit the lowest affinity for Fc-receptors and mediate limited Fc-effector functions [[33], [41]] and may act as an anti-inflammatory or blocking antibody because its high affinity for the *Mtb* antigen competing with other IgG subclasses  [41]. Although IgG4 levels in our patient group did not decline over the course of treatment, ADCC activity showed a modest inverse correlation with IgG4 levels. This may be explained by the ability of elevated IgG4 to compete more effectively for ADCC epitopes in Ag85A, therefore dampening ADCC responses. This is supported by previous studies pointing that elevated IgG4 levels indicate a persistent inflammatory state typically associated with a high bacterial burden. Thus, decreased IgG4 levels at the end of treatment may serve as a marker of successful treatment [17]. Sex and age are well-established drivers of heterogeneity in both humoral and cellular immunity particularly NK cell phenotypes and function [[42], [43]]. Our data analysis demonstrated that the longitudinal resolution of the IgG3 and NK cell signature is a direct reflection of mycobacterial clearance rather than a demographic artifact. This suggests that the intense immunological remodeling driven by active *Mtb* infection and subsequent therapeutic clearance exerts a dominant effect over baseline age- or sex-associated antibody features and NK cell variations. Nonetheless, monitoring these parameters remains crucial for broader multicentric validation studies to ensure generalizability.

Overall, our study investigated an underexplored question- the role of *M. tuberculosis*–specific antibodies in mediating cellular cytotoxicity during treatment, revealing a dynamic and antigen-dependent functional response. These findings provide valuable insights into the identification of immunological biomarkers for therapeutic monitoring and vaccine development in tuberculosis. Importantly, our results highlight the clinical relevance of antibody-based biomarkers, which could complement existing diagnostic approaches and contribute to more precise and personalized patient management.

While this exploratory study provides novel insights, it is subject to certain limitations, most notably a small sample size resulting from patient attrition during follow-up. This reduction in statistical power inevitably restricts the generalizability of our findings, necessitating further validation in larger, adequately powered cohorts. Furthermore, the absence of patient-derived effector cells precluded a direct assessment of cellular immune mechanisms; future research should therefore prioritize the characterization of key cytotoxic markers, such as granzyme A/B and CD107a. Finally, future studies should employ an intracellular CFU (colony former unit) reduction assay using *in vitro* intracellular infection model such as *M. tuberculosis*-infected THP1-macrophages or primary monocytes co-cultured with patient-derived Ag85A-specific antibodies from different treatment timepoints in the presence of autologous or donor NK cells, to directly demonstrate that the ADCC activity observed here translates into mycobacterial killing. This approach is critical because anti-tuberculosis therapy induces dynamic shifts in antibody glycosylation [17]. These treatment-driven modifications dictate affinity for specific FcgR, notably on NK cells, fundamentally altering downstream ADCC and opsonophagocytosis. Integrating this structural remodeling data bridges the gap between longitudinal antibody kinetics and functional pathogen clearance.

## 5 Conclusion

Our findings reveal a functional evolution of the humoral response during anti-TB therapy, where *Mtb*-specific antibodies transition toward an enhanced cytotoxic profile. By the end of treatment, theses antibodies, acting in synergy with NK cells, emerge as pivotal effectors that likely drive bacterial clearance and clinical cure. These antibody-Fc profiles thus emerge as potent functional biomarkers and surrogates of clinical resolution. Ultimately, harnessing this antibody-NK cell axis offers a transformative path for the rational design of next-generation TB vaccines and host-directed immunotherapies.

## Availability of data and materials

All data generated or analyzed during this study are included in this published article and its supplementary information files.

## CRediT authorship contribution statement

**Catherine Sulca:** Writing – original draft, Methodology, Investigation. **Alessia Piamonte:** Writing – original draft, Methodology, Investigation. **María A. Quispe-Ricalde:** Writing – original draft, Conceptualization. **Sandra Delgado:** Writing – review & editing, Writing – original draft, Supervision, Conceptualization. **Sandra Palma Albornoz:** Writing – review & editing, Conceptualization. **Iskra Tuero:** Writing – review & editing, Visualization, Validation, Supervision, Resources, Funding acquisition, Conceptualization.

## Ethics approval and consent to participate

This study was performed in line with the principles of the Declaration of Helsinki. The study was approved by the Institutional Review Board of Universidad Peruana Cayetano Heredia (protocol number 211318) and the DIRIS Lima Centro (protocol number 202364671.

## Declaration of competing interest

The authors declare that they have no known competing financial interests or personal relationships that could have appeared to influence the work reported in this paper.

## Figures and Tables

**Supplementary Figure S1:**
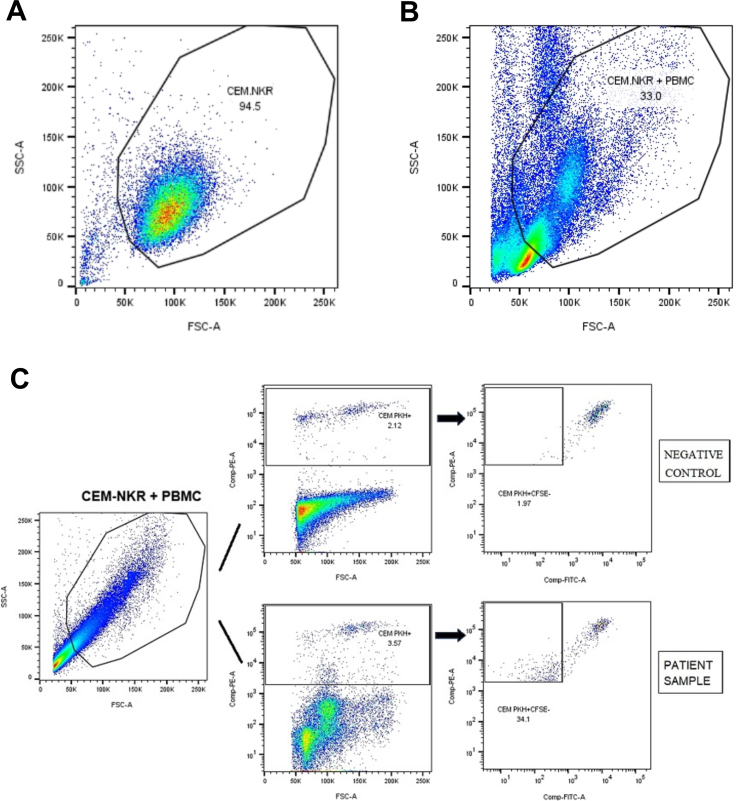
Components of the ADCC response. (A) CEM-NKR cells as a targets. (B) CEM-NKR con cultured with PBMC as effectors. (C) Flow cytometry gating strategy for ADCC. CEM-NKR target cells (5,000 cells) were labeled with PKH-26 (membrane dye, y-axis) and CFSE (intracellular dye, x-axis), and co-cultured with 250,000 PBMC (E:T ratio 50:1). Specific ADCC was defined by the frequency of PKH-26+CFSE- CEM-NKR cells. As a negative control, BSA-coated CEM-NKR cells were used. Representative dot plots are shown in (C).

**Supplementary Figure S2:**
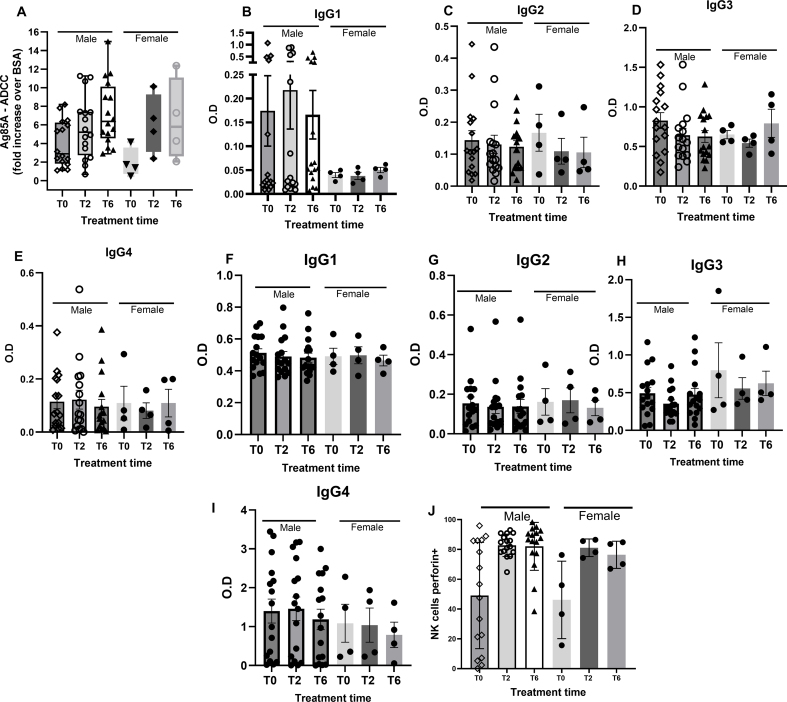
Longitudinal trajectories of Ag85A-specific ADCC scores (A) Plasma levels of Ag85A (B-E) and SARS-CoV2-RBD (F-I) - specific IgG subclasses across treatment by sex. Longitudinal trajectories of NK cell Perforin+ (J) by sex. Results are expressed as mean ± SEM. The differences between male and females were determined using the non-parametric Mann Whitney U test.

**Supplementary Figure S3:**
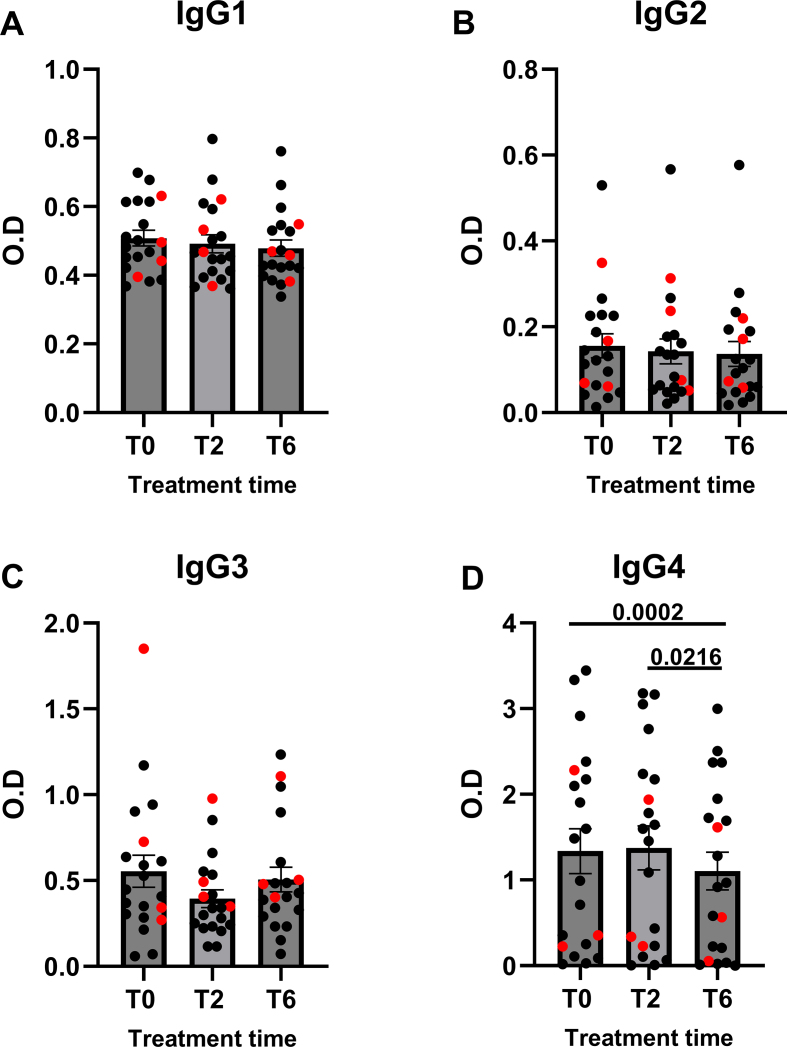
Plasma levels of SARS-CoV2-RBD-specific IgG subclasses across treatment. Longitudinal dynamics of SARS-CoV2 RBD specific IgG1 (A), IgG2 (B), IgG3 (C), and IgG4 (D) were measured in plasma samples from TB patients. In all plots, the y-axis represents the optical density (O.D) values. Results are expressed as mean ± SEM. Data for female participants highlighted in red color. The differences between treatment times were determined using the non-parametric Friedman test and the post hoc statistical Nemenyi test.

**Supplementary Figure S4:**
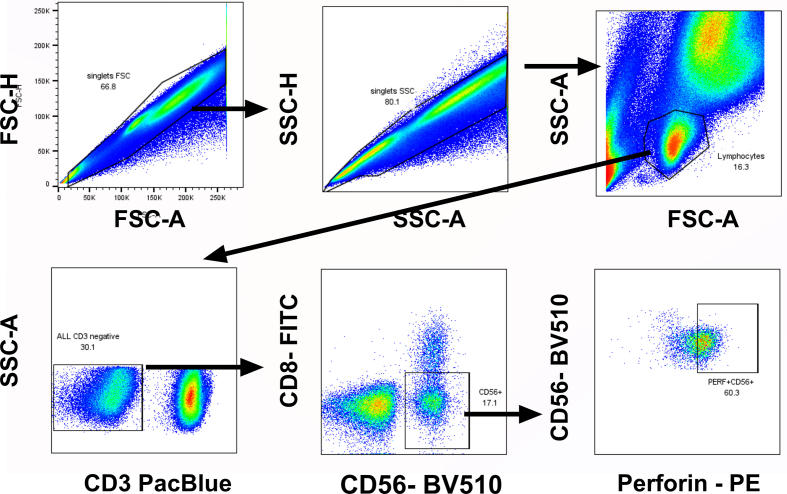
Flow cytometry gating strategy for NK cells characterization in whole blood. Doublets were excluded via FSC-A/ FSC-H and SSC-A/SSC-H plots. Lymphocytes were identified by FSC-A/SSC-A, and NK cells were defined as the CD3-CD8-CD56⁺ population. Withing this compartment, perforin- producing NK cells were quantified using the CD56 versus perforin plot.

## Glossary

ADCC: Antibody-Dependent Cellular Cytotoxicity
ATB: Active Tuberculosis
BCG: Bacillus Calmette-Guérin
BSA: bovine serum albumin
CFSE: Carboxyfluorescein diacetate succinimidyl ester
FBS: Fetal Bovine Serum
Fc: Fragment crystallizable region of antibodies
FcR: Receptors of Fc region of antibodies
HIV: Human immunodeficiency virus
HRP: horse radish peroxidase
IFN-γ: Interferon gamma
kDa: kilodalton
*Mtb*: *Mycobacterium tuberculosis*
NK: Natural Killer cells
PBS: Phosphate Buffered Saline
TB: Tuberculosis

## Appendix A Supplementary data

Supplementary material 5 Correlation between age and immunological parameters in TB patients
