# Supplementary material for: Tuberculosis treatment and cytotoxic immune signatures: a longitudinal study
Source: J Clin Tuberc Other Mycobact Dis. 2026 Jun 23;44:100626. doi: 10.1016/j.jctube.2026.100626 (PMC13333371; doi:10.1016/j.jctube.2026.100626)
Supplement: Supplementary material 5 — Correlation between age and immunological parameters in TB patients [file mmc5.docx]

**Supplementary Table 1**: Correlation between age and immunological parameters in TB patients

| **Comparison** | **r** | **P (two-tailed)** |
| --- | --- | --- |
| Age vs T0 IgG1 Sars-Cov2 | -0.01583 | 0.9472 |
| Age vs T2 IgG1 Sars-Cov2 | -0.007162 | 0.9761 |
| Age vs T6 IgG1 Sars-Cov2 | -0.2767 | 0.2377 |
| Age vs T0 IgG2 Sars-Cov2 | 0.1617 | 0.4958 |
| Age vs T2 IgG2 Sars-Cov2 | 0.007539 | 0.9748 |
| Age vs T6 IgG2 Sars-Cov2 | 0.2479 | 0.2919 |
| Age vs T0 IgG3 Sars-Cov2 | -0.2042 | 0.3878 |
| Age vs T2 IgG3 Sars-Cov2 | -0.1738 | 0.4638 |
| Age vs T6 IgG3 Sars-Cov2 | 0.104 | 0.6625 |
| Age vs T0 IgG4 Sars-Cov2 | 0.2917 | 0.212 |
| Age vs T2 IgG4 Sars-Cov2 | 0.01281 | 0.9572 |
| Age vs T6 IgG4 Sars-Cov2 | 0.2818 | 0.2286 |
| Age vs T0  NKPerf | -0.1349 | 0.5707 |
| Age vs T2 NKperf | -0.08336 | 0.7268 |
| Age vs T6 NKperf | 0.03391 | 0.8871 |
| Age vs T0 ADCC fold | -0.1492 | 0.5301 |
| Age vs T2 ADCC fold | -0.1741 | 0.4629 |
| Age vs T6 ADCC fold | 0.4039 | 0.0774 |
| Age vs T0 IgG1 Ag85 | 0.2839 | 0.2251 |
| Age vs T2 IgG1 Ag85 | 0.06335 | 0.7908 |
| Age vs T6 IgG1 Ag85 | 0.3995 | 0.0809 |
| Age vs T0 IgG2 Ag85 | -0.1327 | 0.5771 |
| Age vs T2 IgG2 Ag85 | -0.1591 | 0.503 |
| Age vs T6 IgG2 Ag85 | 0.1775 | 0.454 |
| Age vs T0 IgG3 Ag85 | 0.1703 | 0.4728 |
| Age vs T2 IgG3 Ag85 | 0.06481 | 0.786 |
| Age vs T6 IgG3 Ag85 | 0.3 | 0.1987 |
| Age vs T0 IgG4 Ag85 | 0.3076 | 0.1871 |
| Age vs T2 IgG4 Ag85 | 0.1756 | 0.459 |
| Age vs T6 IgG4 Ag85 | 0.3158 | 0.1751 |
